# The Effect of Injection Molding Processing Parameters on Chrome-Plated Acrylonitrile Butadiene Styrene-Based Automotive Parts: An Industrial Scale

**DOI:** 10.3390/polym17131787

**Published:** 2025-06-27

**Authors:** Yunus Emre Polat, Mustafa Oksuz, Aysun Ekinci, Murat Ates, Ismail Aydin

**Affiliations:** 1 ALBA Plastic Industry and Trade Inc., TOSB Organized Industrial Zone, 1st Avenue, 13th Street, No. 4, Şekerpınar, 41420 Çayırova, Turkey; yunusemrepolat2468@gmail.com; 2 Department of Polymer Materials Engineering, Faculty of Engineering, Yalova University, 77200 Yalova, Turkey; 3 Faculty of Engineering and Architecture, Recep Tayyip Erdogan University, 53100 Rize, Turkey; 4 Department of Materials and Materials Processing Technologies, Yalova Vocational School, Yalova University, 77200 Yalova, Turkey; aysun.ekinci@yalova.edu.tr; 5 Department of Chemistry, Faculty of Science, Nigde Omer Halisdemir University, 51100 Nigde, Turkey; 6 Department of Chemistry, Faculty of Arts and Sciences, Tekirdag Namik Kemal University, Degirmenalti Campus, 59030 Tekirdag, Turkey; mates@nku.edu.tr; 7 Nanochem Polymer Energy Company, Silahtaraga Mah., University 1st Street, Number: 13/1 Z102, 59860 Tekirdag, Turkey; 8 Rheology Laboratory, Department of Chemical Engineering, Faculty of Engineering, Istanbul University-Cerrahpasa, 34320 Istanbul, Turkey; i.aydin@iuc.edu.tr

**Keywords:** processing parameters, ABS, chrome-plated, adhesion

## Abstract

In recent years, plastic decorative materials have been used in the automotive industry due to their advantages such as being environmentally friendly, aesthetic, light and economically affordable. Plastic decorative materials can exhibit high strength and metallic reflection with metal coatings. Chrome plating is generally preferred in the production of decorative plastic parts in the automotive industry. In this study, the effect of injection molding processing parameters on the metal–polymer adhesion of chrome-plated acrylonitrile butadiene styrene (ABS) was investigated. The ABS-based front grille frames are fabricated by means of using an industrial-scale injection molding machine. Then, the fabricated ABS-based front grille frame was plated with chrome by means of the electroplating method. The metal–polymer adhesion was investigated as a function of the injection molding processing parameters by means of a cross-cut test and scanning electron microscope (SEM). As a result, it was determined that the optimal injection process parameters, a cooling time of 18 s, a mold temperature of 70 °C, injection rates of 45-22-22-20-15-10 mm/s, and packing pressures of 110-100-100 bar, were effective in enhancing polymer–metal adhesion for the ABS-based front grille frame.

## 1. Introduction

In recent years, plastic materials have seen great interest in the automotive industry due to their unique properties such as lightness [1,2], low cost [3,4], recycling capabilities [5,6], environmental friendliness [7,8], corrosion resistance [9,10], ease of processing [11,12] and toughness [13,14]. This is especially the case in the many parts of a vehicle produced from plastic raw materials due to its aesthetic appearance such as the logo, handles, panels and buttons, etc. [15,16]. Automotive plastics are produced by means of an injection molding process with a complex geometry such as differing length, width, height and thickness, etc. [17,18]. In addition, plastic parts are given a decorative appearnace by means of chrome plating with the electroplating technique [19,20]. The chrome plating of the plastic parts provides brightness and visuality like a metallic surface [21,22]. Generally, acrylonitrile butadiene styrene (ABS) is widely used in the chrome electroplating process and is a versatile engineering thermoplastic [23,24]. ABS is used widely for the fabrication of automotive parts such as grilles, door handles and trim, exterior components, and electrical components due to its excellent adhesion properties [25,26], toughness [27,28], dimensional stability [29,30] and good chemical resistance [31,32]. In the coating process, firstly, the molded ABS surface is etched, creating small, microscopic voids on its surface; thanks to the small gaps created in the plastic, the metal layer is adhered to the plastic surface [33,34].

Seo et al. [35] reported that the depths on the ABS surface are important in terms of metal coating performance. Ozcelik et al. [36] investigated the effect of injection pressure and cooling system on the properties of an ABS component in automotive components during the injection molding process. It has been reported that the optimization of injection molding processing parameters will be effective in preventing surface defects of ABS parts due to shrinkage. Schiffersa et al. [37] investigated the surface properties of ABS and polycarbonate/ABS parts after etching by applying electroplating. They reported that the etching process applied before the coating was effective in the shrinkage of the coating layers, and that the coating quality of chrome-plated ABS and polycarbonate/ABS parts was determined. Kurek et al. [38] fabricated poly (vinyl chloride) (PVC) and PVC/ABS blends by means of the injection molding process. Then, the fabricated plastic parts were chrome plated using the conventional method. The surfaces of the PVC/ABS blends and PVC samples were analyzed by scanning electron microscope (SEM), atomic force microscope (AFM) and contact angle. It has been reported that the surface roughness of the plastic part directly affects the adhesion of the metal layer to the polymer surface. Karabeyoglu et al. [39] investigated the effect of the wear behavior of ABS samples produced by injection molding on the coating performance. ABS samples were coated with Copper–Chrome–Nickel (Cu–Ni–Cr) after injection molding. The average friction coefficient and friction forces, surface roughness and wear level of the coated and uncoated samples were determined with a profilometer. They found that the multilayer Cu–Ni–Cr coating significantly affects the friction forces and the average friction coefficient values. Sreedharan et al. [40] investigated the effects of the plastic injection, mold and coating parameters on k metal coating thicknesses. Injection molding optimization of an ABS-based automotive exterior part manufacturing was obtained by industrial measurements. They found that injection molding processing parameters such as filling time, mold temperature, melt temperature, holding pressure, and holding time in an injection-molded plastic part are important in determining the coating performance. Oliveira et al. [41] evaluated the surface morphology, roughness and gloss of injection-molded ABS parts in relation to the injection molding processing parameters. It has been observed that the surface properties are affected by injection molding processing parameters such as mold temperature, injection temperature and holding pressure. Monterde et al. [42] investigated the effect of injection molding processing parameters on the surface roughness of ABS foams. They determined that the mold temperature and injection rate, which are the injection molding processing parameters, are effective on the surface roughness of ABS.

The coating performance of chrome-plated plastics varies depending on the structural–physical properties of the plastics and the processing parameters, as well as mechanical locking, electrostatic, diffusion, adsorption and chemical bonding [43,44]. The optimization of the injection parameters (injection pressure, melt temperature, mold temperature and injection time) affects the surface properties of the plastic part [45,46]. Since chrome plating is applied to the surface of the plastic part, the parameters of the injection molding process directly affect the metal–polymer adhesion [47,48]. Residual stresses can occur on the plastic surface due to effects such as temperature and pressure in the complex process stages during the injection molding of ABS. Residual stresses directly affect the quality and performance of injection-molded parts. If residual stresses occur in ABS parts, some problems such as bending, cracks, breaks and double fractures can occur. Coating metals have difficulty adhering to or cannot adhere to areas where residual stress occurs after injection molding in ABS parts.

We aimed to solve the coating problems in chrome-plated ABS parts by optimizing the injection molding parameters, which are more industrially practical, rather than solving them with long-term and costly plating bath formulation or electroplating parameter changes. The final product quality and process efficiency of ABS parts are affected by the injection molding processing parameters [49,50]. Especially in plastic decorative automotive parts, its aesthetic appearance is of crucial importance [51,52]. Several studies in the literature have focused on optimizing electroplating coating parameters and changing chrome plating bath formulations. In this study, the effect of injection molding processing parameters (cooling time, injection rate, packing pressure and mold temperature) on the metal–polymer adhesion performance of chrome-plated ABS-based decorative automotive parts after injection molding was investigated. The ABS-based front grille frame of a commercial van was fabricated on an industrial scale by means of the injection molding process. Then, the fabricated ABS-based front grille frame of a commercial van was chrome plated using the industrial electroplating process. The effects of the injection molding processing parameters on the coating performance of chrome-plated ABS parts were determined in terms of cooling time, injection rate, packing pressure and mold temperature. The metal–polymer adhesion performance of the chrome-plated ABS-based front grille frame as a function of the injection molding processing parameters were examined by means of scanning electron microscope (SEM), optical microscope, cross-cut test and coating thickness measurement.

## 2. Materials and Methods

### 2.1. Materials

Unfilled acrylonitrile butadiene styrene granules with the trade name Novodur^®^ P2MC were supplied from INEOS Styrolution Inc. (Ludwigshafen, Germany) which is an injection molding material and suitable for electroplating. The impact properties of ABS are Charpy impact strength value of 150 kJ/m^2^ at −30 °C and Charpy notched impact strength value of 25 kJ/m^2^ at +23 °C, and 16 kJ/m^2^ at −30 °C, all measured according to ISO 179. The density value of ABS is 1.03 g/cm^3^, melt flow index (MFI) value of 25 g/10 min under 220 °C at 10 kg load according to ISO 1133 standard, Vicat softening temperature (VST) value of 97.5 °C under 50 N at 120 °C/h according to ISO 306 standard and linear mold shrinkage value of 0.2–0.7% according to ISO 294-4 standard.

### 2.2. Fabrication of Chrome-Plated ABS-Based Automotive Parts

**Figure 1:**
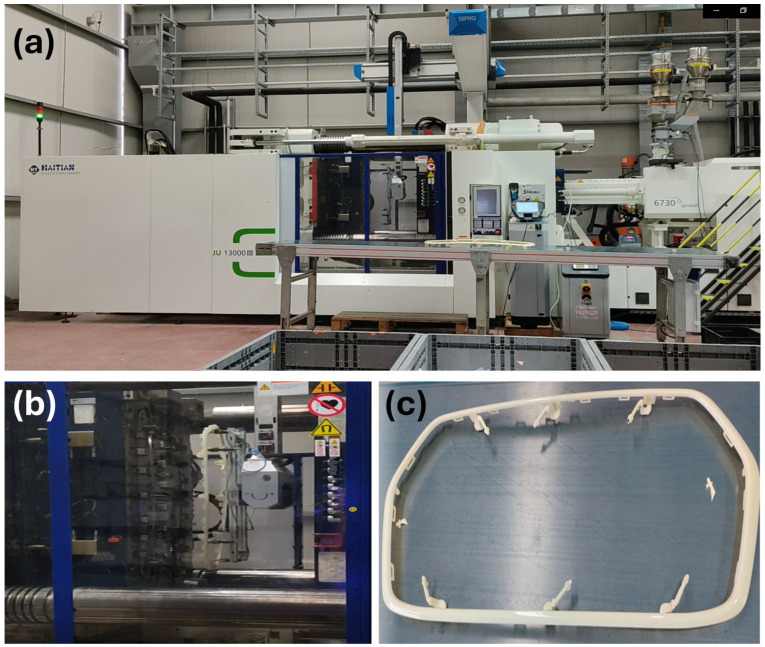
(**a**) The injection molding machine, (**b**) injection mold of the ABS-based front grille frame and (**c**) fabricated ABS-based front grille frame.

Before the injection molding process, ABS raw material was dried at 90 °C for 4 h by means of MOTON Luxor S 80 model dryer. The molding process of the ABS parts were performed by means of a Haitian JU13000III model injection molding machine. In the fabrication of the ABS-based automotive parts, eight in-line hot runner molds made of 1.2343 type steel were used. In all of the automotive parts, the composition of ABS materials content is the same and only the injection molding processing parameters have been changed during injection molding in terms of cooling time, injection rate, packing pressure and mold temperature. The injection molding processing parameters (cooling time, injection rate, packing pressure and mold temperature) are given in Table 1. According to the unfilled ABS granules supplier’s datasheet, the injection molding process melt temperature values are 230-230-230-225-220-220-210-210 °C. The same injection temperature profile was used for all samples during the injection molding process. The injection molding temperature profile used in our study was determined by considering the melting point, viscosity behavior and thermal degradation limits of the ABS material used. In selecting the profile, the aim was to ensure homogeneous flow of the polymer within the mold and sufficient mold filling during injection to optimize processability. The injection molding machine, injection mold of the ABS-based front grille frame and fabricated ABS-based front grille frame are given in Figure 1.

**Figure 2:**
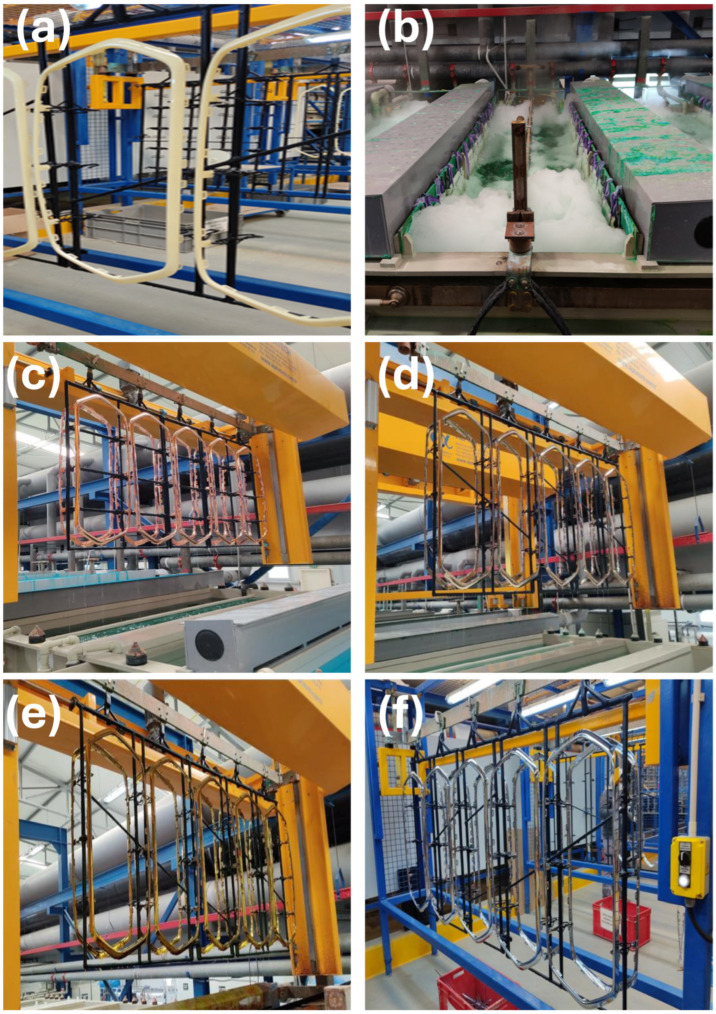
(**a**) Chrome-unplated ABS-based front grille frame, (**b**) chrome–sulfuric acid bath, (**c**) copper-plated ABS-based front grille frame, (**d**) nickel-plated ABS-based front grille frame, (**e**) ABS-based front grille frame after chrome electrodeposition bath and (**f**) chrome-plated ABS-based front grille frame.

The chrome electroplating process consisted of multiple layers of copper, nickel, and chrome. Injection-molded ABS-based automotive parts were chrome plated with a chrome plating process, through a series of processes consisting of 71 plating bath steps: 1. washing with deionized water at 25 °C, 2–5. etching, 6–8. washing with deionized water at 25 °C, 9. chromium reduction (hexavalent chromium is reduced to trivalent chromium), 10. washing with deionized water at 25 °C, 11. ion exchange water, 12. pre-dip, 13–14. activator, 15–16. washing with deionized water at 25 °C, 17. ion exchange water, 18. accelerator, 19–20. washing with deionized water at 25 °C, 21. ion exchange water, 22–25. electroless nickel, 26–27. washing with deionized water at 25 °C, 28. ion exchange water, 29–30. pre-nickel (electric current is applied to the bath), 31–32. washing with deionized water at 25 °C, 33. ion exchange water, 34–41. acidic copper (electric current is applied to the bath), 42–43. Washing with deionized water at 25 °C, 44. ion exchange water, 45. copper activation, 46–47. washing with deionized water at 25 °C, 48. ion exchange water, 49–51. semi-bright nickel (electric current is applied to the bath), 52–54. bright nickel (electric current is applied to the bath), 55–56. washing with deionized water at 25 °C, 57. ion exchange water, 58. microcracked nickel (electric current is applied to the bath), 59–60. washing with deionized water at 25 °C, 61. chrome activation (electric current is applied to the bath), 62–63. chrome bath, 63–68. washing via deionized water at 25 °C, 69–70. ion exchange water, 71. washing with deionized water at 85 °C. A chrome–sulfuric acid bath was first used to oxidize and remove the butadiene rubber phase on the ABS’ surface. During this process, the ABS’ surface was made porous. An aqueous palladium–tin colloid (Pd particles surrounded by SnCl_2_) was deposited in microscopic holes created by etching the shell. This step creates catalytic sites for the electrolysis layer to grow [26]. The tin chloride shell of the activator colloid was then removed, leaving Pd particles at the etching sites as catalysts. In this step, a thin, sticky metallic film is formed on the surface of the part by chemical reduction. This layer makes the surface conductive for subsequent electroplating processes. The chrome plating process of the ABS-based automotive parts is given in Figure 2.

In order to determine the effect of injection parameters on the adhesion performance of the chrome coating, the ABS-based automotive parts were subjected to the thermally artificial aging process. The thermally artificial aging process was applied to the ABS-based automotive parts with chrome-plated surfaces. The surfaces of the ABS samples were washed with deionized water and dried at room temperature before the thermally artificial aging process. The thermally artificial aging process was carried out in Nucleon, NKT-900 model using a thermal cabinet. After the parts were placed in the cabinet, the parts were exposed to −40 ± 2 °C cold air for 60 min in 1 cycle. The parts were then exposed to hot air at 80 ± 2 °C for 60 min. Overall, 10 repetitive thermal cycles were applied to each part under the same test conditions.

### 2.3. Characterization

The microcracking density measurements on the surfaces of the ABS-based automotive parts were performed by means of optical microscope. A ZEIZZ, AX10 model optical microscope was used. The ABS surface images were obtained at a magnification of approximately 500 µm. The microcrack density of the samples was calculated by means of computer software with linear lines drawn on the optical microscope images.

**Figure 3:**
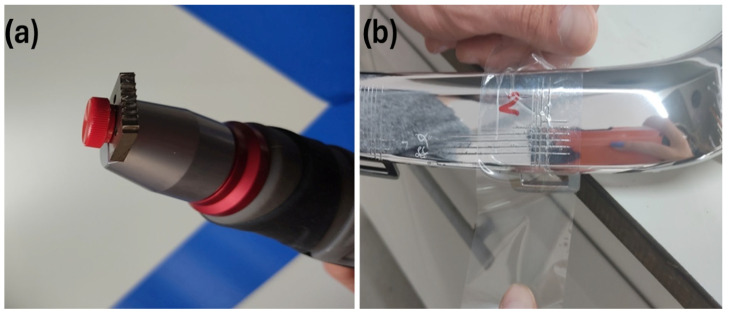
(**a**) Cross-cut knife and (**b**) cross-cut applied to an ABS-based automotive part.

The cross-cut tests were performed on aged and unaged chrome-plated ABS-based automotive parts according to ISO 2409 standards at room temperature. The TQC sheen, SP1691 model cross-cut equipment was used. The cross-cut equipment has a multi-knife cutter with 2 mm spacing and 6 teeth. Firstly, horizontal scratches were applied to the surface of the plastic part using the cross-cut knife at a 90⁰ angle. Then, vertical scratches were applied on top of the horizontal scratches using the cross-cut knife at a 90⁰ angle. Thus, a lattice structure was formed on the surface of the parts. In the last step, a special tape was adhered to the created cage area and pulled off with constant force. The cross-cut knife and tested sample are given in Figure 3.

The coating thickness of the ABS-based automotive parts was measured using an electrochemical method by means of a Fischer/CMS2 STEP model Couloscope. F4, F6 and F1 electrolytes were supplied from Yalcın Galvano Chemistry Inc and used for thickness measurements. In order to measure the thickness of the coating, three different electrodes were dropped on the coated ABS parts, separately. The F4 electrolyte was used to measure the thickness of the copper layer. The F6 electrolyte was used to measure the thickness of the nickel layer. The F1 electrolyte was used to measure the thickness of the chrome layer. The sum of the copper, chromium and nickel layer thicknesses were recorded to determine the total thickness of the coating layer.

The sample surfaces were analyzed using an electron microscope after treatment with glacial acetic acid. The parts were first immersed in a cold acetic acid bath for 30 s. They were then rinsed with water and dried at room temperature. Subsequently, they were re-immersed in the cold acetic acid bath for 120 s, rinsed again with water, and dried at room temperature. The data obtained from the imaging were used to evaluate surface residual stresses for each set of injection process parameters. The scanning electron microscope (SEM) analysis was performed for the etched ABS-based automotive parts before chrome plating and the chrome-plated ABS-based automotive parts after the aging process. A Zeiss Supra 40 VP branded SEM device equipped with energy dispersive X-ray analysis (EDX) was used in the analysis. The surfaces of all the samples were coated with gold–palladium (Au–Pd) before SEM analysis.

## 3. Results

### 3.1. Microcrack Density

**Figure 4:**
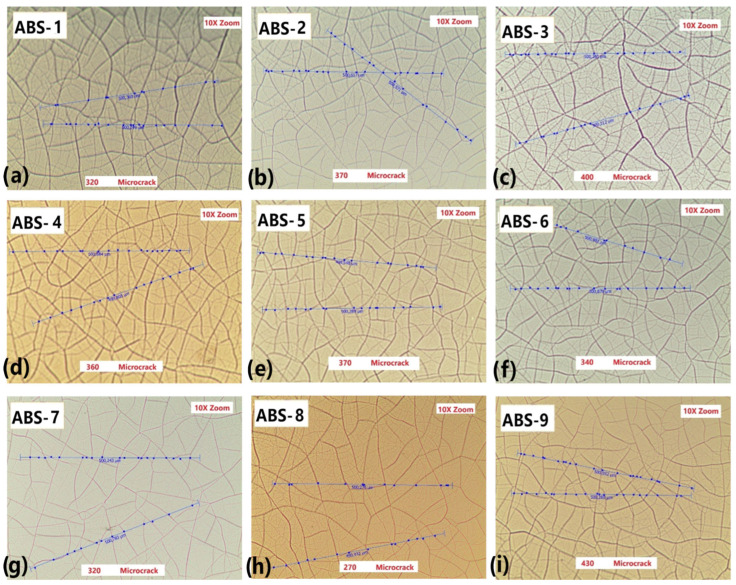
Optical microscope images of aged (**a**) ABS–1, (**b**) ABS–2, (**c**) ABS–3, (**d**) ABS–4, (**e**) ABS–5, (**f**) ABS–6, (**g**) ABS–7, (**h**) ABS–8 and (**i**) ABS–9.

The effect of the injection molding processing parameters on microcrack density were investigated. Microcrack density is a key parameter for hard chrome electroplating. Microcrack density greatly affects the behavior of the chemical and mechanical properties of the chrome-plated ABS-based automotive parts. The optical microscope images of microcracking density on the surfaces of the ABS parts are given in Figure 4. The microcrack density of the samples was obtained as 320 grains for ABS-1 (Figure 4a), 370 grains for ABS-2 (Figure 4b), 400 grains for ABS-3 (Figure 4c), 360 grains for ABS-4 (Figure 4d), 370 grains for ABS-5 (Figure 4e), 340 grains for ABS-6 (Figure 4f), 320 grains for ABS-7 (Figure 4g), 270 grains for ABS-8 (Figure 4h) and 430 grains for ABS-9 (Figure 4i). In the ABS-2 sample, where the fixed half-mold temperature was decreased by 15 °C, the number of observed microcracks was higher compared with the ABS-1 sample, in which the fixed half-mold temperature was increased by 15 °C. This indicates that increasing the fixed half-mold temperature has a favorable effect on reducing microcrack formation. In the ABS-3 sample, it was observed that increasing the packing pressure by 20 bars also contributed to a reduction in microcrack formation. For the ABS-6 sample, increasing the injection rate by 5 mm/s at each of the stages resulted in a higher number of microcracks compared with the ABS-5 sample, where the injection rate was decreased by 5 mm/s at each stage. These findings suggest that reducing the injection rate has a beneficial effect on minimizing microcrack formation. Similarly, the number of microcracks in the ABS-8 sample, where the cooling time was shortened by 7 s, was higher than in the ABS-7 sample, where the cooling time was extended by the same amount. It was concluded that increasing the cooling time also contributes to reducing microcrack formation.

It is seen that the microcrack density of the samples changed as a function of the injection molding processing parameters. The variation in microcrack density among the samples is indicative of differences in internal stress relaxation mechanisms during the electroplating process. A moderate microcrack density is generally desirable to alleviate residual stress without excessively compromising the barrier properties of the coating. On the other hand, samples such as ABS-8, exhibiting lower microcrack density, may be more prone to brittle failure under mechanical or thermal stress. Therefore, the careful control of microcrack formation is essential for optimizing the performance and longevity of chrome coatings on ABS.

### 3.2. Coating Thickness Measurement

The coating thickness of the ABS-based automotive parts was determined by measuring the coating thicknesses of the copper, nickel and chrome layers separately. The coating layer thickness values of the samples are given in Table 2. It is seen that the coating layer thickness values change as a function of the injection molding processing parameters. It is seen that the chrome plating thickness is thinner than the copper and nickel layer thicknesses. It is seen that the nickel layer thickness values of the samples are changed from 17.9 to 23.7 µm. It is seen that the copper layer thickness values of the samples are changed from 21.0 to 24.7 µm. The electroplated chrome layer was obtained as 1.65 ± 0.4 µm. These coating thickness values are in the same range as the total coating layer thicknesses of the electroplated ABS-6, ABS-7, ABS-8 and ABS-9 samples. The highest thickness of total coating layer was obtained as 48.5 µm in the ABS-5 sample. Injection-molded plastic parts with complex geometries, increased stress levels and increased metal layer thickness can be more difficult to coat [34,35]. Ensuring consistent multilayer coating deposition is of critical importance for achieving optimal performance, enhanced reliability, and prolonged service life of chrome-plated ABS in practical industrial applications.

### 3.3. Cross-Cut Test

**Figure 5:**
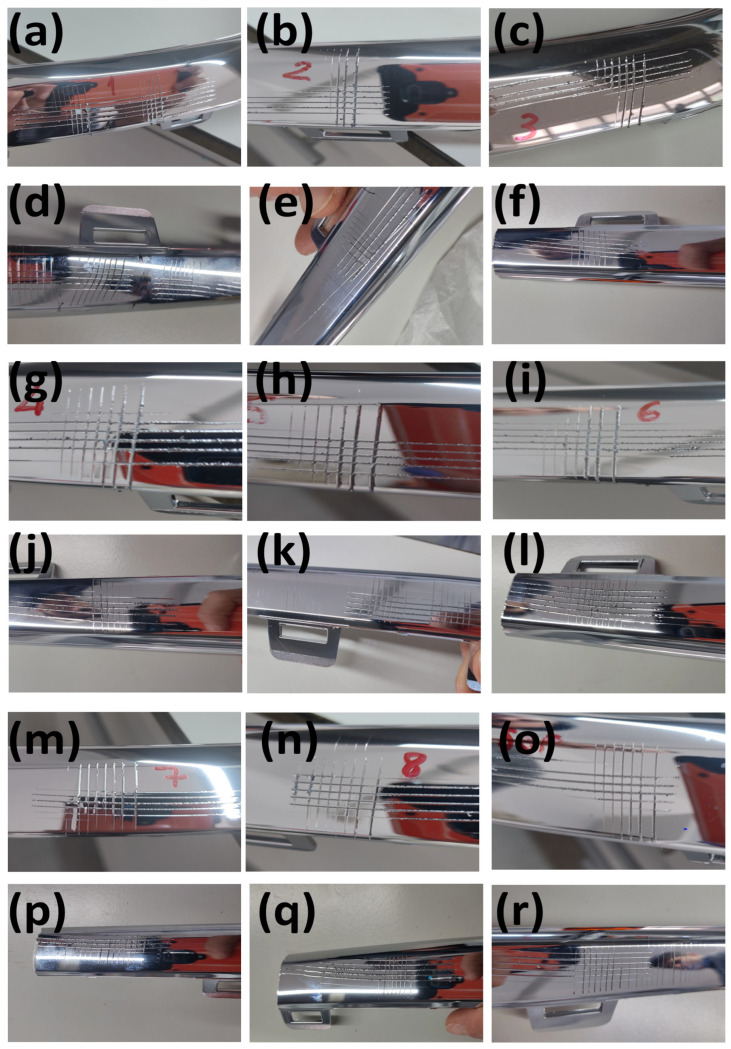
Images of the cross-cut test applied to (**a**) unaged ABS–1, (**b**) unaged ABS–2, (**c**) unaged ABS–3, (**d**) aged ABS–1, (**e**) aged ABS–2, (**f**) aged ABS–3, (**g**) unaged ABS–4, (**h**) unaged ABS–5, (**i**) unaged ABS–6, (**j**) aged ABS–4, (**k**) aged ABS–5, (**l**) aged ABS–6, (**m**) unaged ABS–7, (**n**) unaged ABS–8, (**o**) unaged ABS–9, (**p**) aged ABS–7, (**q**) aged ABS–8 and (**r**) aged ABS–9 samples.

The effect of injection molding processing parameters on coating performance were investigated by means of a cross-cut test. The aged and unaged chrome-plated ABS-based automotive parts after the cross-cut test are given in Figure 5. It is seen that the adhesion of the metallic layer on the ABS plastic surface changed as a function of the injection molding processing parameters. The number of blocks removed was an indication of the adhesion of the metallic layer onto the ABS surface.

Although increasing the fixed half-mold temperature by 15 °C in the ABS-1 sample and reducing the packing pressures by 20 bars at each stage (a total of three stages) in ABS-4 contributed to a reduction in residual stress, both conditions led to minor surface sink marks. As a result, these surface imperfections negatively affected the cross-cut test performance, yielding poorer results compared with the ABS-2 and ABS-3 samples. In ABS-6, increasing the injection rate by 5 mm/s at each of the stages led to higher molecular orientation and increased residual stress on the part’s surface, which resulted in inferior performance relative to the ABS-5 sample. Similarly, in ABS-8, reducing the cooling time by 7 s increased residual stress on the part’s surface, leading to worse outcomes in comparison with the ABS-7 sample. This adhesion is quite acceptable for the all unaged (Figure 5a–c,g–i,m–o) and unaged (Figure 5d–f,j–l,p–r) ABS-based automotive parts demonstrating good metal–polymer adhesion. Furthermore, it was observed that the injection process parameters employed in this study positively influenced the surface quality of the produced ABS parts. Therefore, the selected process parameters are considered to provide optimal values in terms of both material properties and process stability.

A comparison of cross-cut test results from the literature is given in Table 3. Kurek et al. [47] investigated chrome-plated ABS, with the corresponding cross-cut test image demonstrating minimal coating detachment, indicating excellent adhesion. Yudhanto et al. [33] investigated copper-plated ABS, where the tape test results reveal substantial coating removal, corresponding to a 1B adhesion rating and reflecting poor interfacial bonding. Alhamad et al. [53] investigated NiP-TiC-SiC-plated ABS, with sequential images demonstrating varying degrees of coating integrity, suggesting moderate to good adhesion depending on the deposition conditions. Kim et al. [54] investigated copper-plated ABS, with the cross-cut images demonstrating the progressive deterioration of adhesion quality across different test conditions.

### 3.4. SEM Analysis

**Figure 6:**
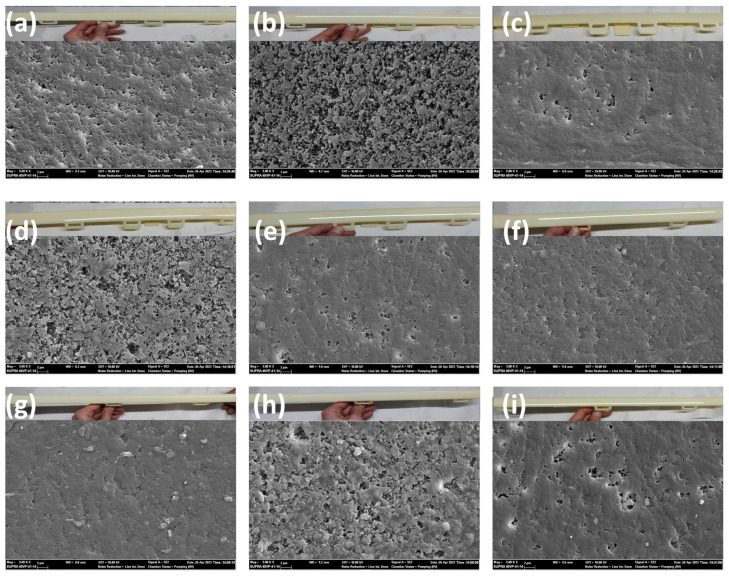
SEM micrographs of chrome-unplated (**a**) ABS–1, (**b**) ABS–2, (**c**) ABS–3, (**d**) ABS–4, (**e**) ABS–5, (**f**) ABS–6, (**g**) ABS–7, (**h**) ABS–8 and (**i**) ABS–9.

The effect of the injection molding processing parameters on metal-polymer adhesion in ABS-based automotive parts coated with chrome after injection molding was investigated by SEM analysis. The influence of the injection parameters on surface morphology was assessed following acid treatment. The acid-treated samples are shown in Figure 6. The technique employed to detect the residual stresses induced by the injection molding process was based on the experimental observation of whitening on the sample surfaces. In the acetic acid test, the presence of whitening indicates residual stress within the material. Consequently, since no whitening was observed on the sample surfaces, it was determined that residual stresses were absent.

The SEM micrographs of chrome-unplated ABS-based automotive parts are given in Figure 6. The absence of any white appearance on the surface of the ABS parts indicates that no residual stress occurs during injection molding processing. The ABS parts exhibited different morphological behavior depending on the injection molding processing parameters. During the etching step, butadiene in the ABS’s surface was removed, creating voids for mechanical bonding. Mechanical bonding performance in ABS-based automotive parts is determined by the shape, amount and distribution of voids on the surface, polymer formulation, machining and mold geometry [38,39]. For a good chrome plating performance, the surface properties of the polymer are very important for decorative automotive applications [41].

**Figure 7:**
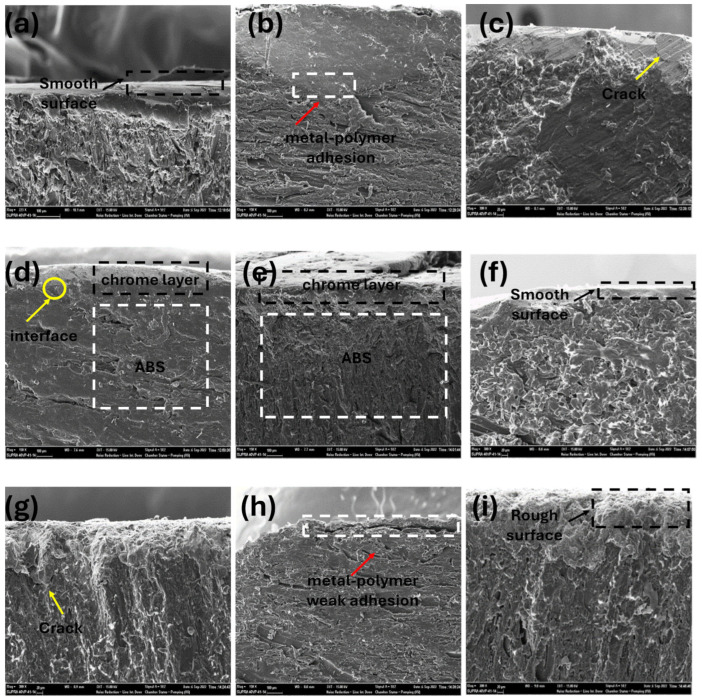
SEM micrographs of chrome-plated (**a**) ABS-1, (**b**) ABS-2, (**c**) ABS-3, (**d**) ABS-4, (**e**) ABS-5, (**f**) ABS-6, (**g**) ABS-7, (**h**) ABS-8 and (**i**) ABS-9 samples.

Thermally artificial aging, often referred to as accelerated thermal aging, is employed to simulate the long-term environmental effects on ABS/chrome adhesion within a shortened timeframe. During this process, coated ABS samples are exposed to elevated temperatures, typically above ambient conditions, to accelerate the physical and chemical degradation mechanisms that would occur over extended periods. These effects result in increased interfacial stresses, microcrack formation and potential delamination at the metal-polymer interface. The SEM micrographs of a cross section of aged chrome-plated ABS-based automotive parts are given in Figure 7. In ABS-1 and ABS-2 (Figure 7a,b), there is a compatibility between ABS and the coating layer. In other words, there appears to be no gap between the polymer and the coating at the interface between the ABS and the electroplated layer. In ABS-3 (Figure 7c), it is seen that there is no gap between the polymer and the coating at the interface between the ABS and the chrome layer. Despite this, cracks formed in the coating layer. Microcracks form between the ABS and the chrome plating layer of ABS-4 (Figure 7d), but no cracks are transmitted to the plating surface. There is a clear match between the ABS and the coating layer of ABS-5 and ABS-6 (Figure 7e,f). In other words, there appears to be no gap between the polymer and the coating at the interface between the ABS and chrome plating. The surface of the coatings is quite smooth. It is seen that there is no gap between the ABS and the chrome plating layer of ABS-7 (Figure 7g). However, microcracks occur in the coating layer and crack propagation occurs at the coating surface. It is seen that there is a gap between the polymer and the coating at the interface between the ABS and the chrome layer of ABS-8 (Figure 7h). It exhibits a rough coating layer which presented the lowest microcrack density (270 microcracks) (Figure 4h). This shows that the injection parameter is not suitable for good polymer–metal adhesion. Microcracks occur in the chrome plating layer of ABS-9 (Figure 7i). There is crack propagation on the coating surface. As a result, good adhesion between the ABS–chrome coating and non-cracked coating is obtained for the samples. The injection parameters of these samples represent the optimum injection molding processing parameters for good metal–polymer adhesion. In conclusion, the integrated microcrack density and SEM analysis reveal that achieving an optimal balance among microcrack density, interfacial morphology and surface quality is essential for ensuring durable and reliable chrome coating on ABS. Microcrack density is a critical parameter in chrome electroplating processes, directly influencing the coating’s mechanical properties, corrosion resistance and overall durability. Chrome coatings naturally develop a network of fine microcracks during deposition and subsequent cooling due to intrinsic stresses arising from the electroplating process. Excessively high microcrack densities or poor adhesion characteristics significantly deteriorate the mechanical integrity and corrosion resistance of the chrome coating on ABS.

## 4. Conclusions

In this study, the effect of injection molding processing parameters on polymer–metal adhesion in chrome-plated ABS-based decorative automotive parts was investigated. Increasing the temperature of the fixed half of the mold from 70 °C to 85 °C weakened the adhesion ability of the coating. Lowering the temperature of the fixed half of the mold from 70 °C to 65 °C had a positive effect on the adhesion of the coating. Increasing the pressure was effective in improving the adhesion ability. Reducing the holding pressure decreased the adhesion ability of the coating. Reducing the injection rate had a positive effect on the adhesion ability of the coating. Increasing the injection rate had a negative effect on the adhesion ability of the coating. Increasing the cooling time from 25 to 32 s increased the adhesion strength of the coating. Reducing the cooling time from 25 to 18 s decreased the adhesion strength of the coating; this was the case where the coating strength was the weakest. A controlled level of microcrack density can be beneficial, as it allows for the relief of internal stress and helps to prevent catastrophic coating failure. Therefore, optimizing microcrack density is essential to balance mechanical robustness with corrosion protection, making it a fundamental consideration in the design and quality assessment of chrome electroplated layers. Thermally artificial aging provides valuable predictive insights into the durability and reliability of ABS chrome coating under service conditions, and it is an essential step in qualifying such materials for long-term applications. The injection parameters were optimized based on the ABS parts that exhibited the best coating performance to form fine pores and cracks.

## Figures and Tables

**Table 1:** Injection molding processing parameters of the parts.

Sample	Cooling Time (s)	Injection Rate (mm/s)	Packing Pressure (bar)	Mold Temperature (°C)
ABS-1	25	45-22-22-20-15-10	110-100-100	85
ABS-2	25	45-22-22-20-15-10	110-100-100	55
ABS-3	25	45-22-22-20-15-10	130-120-120	70
ABS-4	25	45-22-22-20-15-10	90-80-80	70
ABS-5	25	40-17-17-15-10-5	110-100-100	70
ABS-6	25	50-27-27-25-20-15	110-100-100	70
ABS-7	32	45-22-22-20-15-10	110-100-100	70
ABS-8	18	45-22-22-20-15-10	110-100-100	70
ABS-9	25	45-22-22-20-15-10	110-100-100	70

**Table 2:** The coating layer thickness values of the samples.

Sample	Thickness of Chrome Layer (µm)	Thickness of Nickel Layer (µm)	Thickness of Copper Layer (µm)	Thickness of Total Coating Layer (µm)
ABS-1	1.7	21.6	24.3	47.6
ABS-2	2.1	17.9	23.0	43.0
ABS-3	1.9	21.3	24.7	47.9
ABS-4	1.4	19.2	21.5	42.1
ABS-5	1.8	23.3	23.5	48.6
ABS-6	1.5	21.5	23.6	46.6
ABS-7	1.5	20.7	24.1	46.3
ABS-8	1.7	20.8	23.4	45.9
ABS-9	1.3	23.7	21.0	46.0

**Table 3:** Comparison of cross-cut test results from the literature. Reprinted with permission from Refs [33,53,54]. Copyright@Elsevier [33,53,54]. In addition, Reprinted with permission from Ref [47]. Copyright@Wiley [47].

Reference	Polymer/Metal Coating	Image of After Cross-Cut Test
Kurek et al. [47]	ABS/Chrome	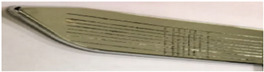
Yudhanto et al. [33]	ABS/Copper	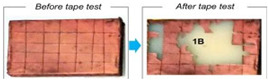
Alhamad et al. [53]	ABS/NiP-TiC-SiC	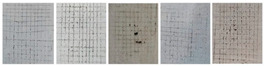
Kim et al. [54]	ABS/Copper	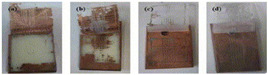

## Data Availability

Not applicable.
